# Prospecting Agro-waste Cocktail: Supplementation for Cellulase Production by a Newly Isolated Thermophilic *B. licheniformis* 2D55

**DOI:** 10.1007/s12010-017-2401-z

**Published:** 2017-02-07

**Authors:** Muinat Olanike Kazeem, Umi Kalsom Md Shah, Azhari Samsu Baharuddin, Nor’ Aini AbdulRahman

**Affiliations:** 1 Department of Bioprocess Technology, Faculty of Biotechnology and Biomolecular Sciences, Universiti Putra Malaysia, 43400 Serdang, Selangor Malaysia; 2 Department of Microbiology, Faculty of Life Sciences, University of Ilorin, Ilorin, Kwara State 1515 Nigeria; 3 Department of Process and Food Engineering, Faculty of Engineering, Universiti Putra Malaysia, 43400 UPM, Serdang, Selangor Malaysia

**Keywords:** Composting, Thermophilic bacteria, Cellulase production, Agro-waste cocktail, Enzyme location, NaOH pretreatment, Scanning electron micrograph (SEM)

## Abstract

Bacteria isolated from thermophilic environment that can produce cellulase as well as utilise agro-waste biomass have a high potential for developing thermostable cellulase required in the biofuel industry. The cost for cellulase represents a significant challenge in converting lignocellulose to fermentable sugars for biofuel production. Among three potential bacteria examined, *Bacillus licheniformis* 2D55 (accession no. KT799651) was found to produce the highest cellulolytic activity (CMCase 0.33 U/mL and FPase 0.09 U/mL) at 18–24 h fermentation when grown on microcrystalline cellulose (MCC) as a carbon source in shake flask at 50 °C. Cellulase production process was further conducted on the untreated and NaOH pretreated rice straw (RS), rice husk (RH), sugarcane bagasse (BAG) and empty fruit bunch (EFB). Untreated BAG produced the highest FPase (0.160 U/mL), while the highest CMCase (0.150 U/mL) was supported on the pretreated RH. The mixture of untreated BAG and pretreated RH as agro-waste cocktail has remarkably improved CMCase (3.7- and 1.4-fold) and FPase (2.5- and 11.5-fold) compared to the untreated BAG and pretreated RH, respectively. The mechanism of cellulase production explored through SEM analysis and the location of cellulase enzymes of the isolate was also presented. Agro-waste cocktail supplementation provides an alternative method for an efficient production of cellulase.

## Introduction

Each year, there are millions of tonnes of lignocellulosic wastes being generated from the agricultural, agro-industrial and forestry industries, which pose a major disposal problem. On the other hand, Malaysia is very lucky to have rice straw, rice husk, oil empty fruit bunch and sugarcane bagasse as the major wastes generated from industrial boilers [1, 2]. Agro-waste material or lignocellulose consists of cellulose and hemicellulose bound together by the lignin sheath. The cellulose and hemicellulose content in agro-waste materials intended to be transformed into value-added products including biosugar, biocompost, biofuels, biochar, biocomposite and additives either through microbial fermentation, thermochemical or enzymatic process is very significant in searching for a new biological resource.

Cellulase is very crucial for biosugar in producing bioethanol due to the recalcitrant and heterologous nature of lignocellulosic materials. Cellulase hydrolyses the β-1,4-d-glucan linkages of cellulose to liberate cello-oligosaccharide, cellobiose and glucose as its major end products. These products are liberated as the result of three enzymes, namely endoglucanase (EG), which exposes the reducing and non-reducing ends of cellulose through incisions, cellobiohydrolase (EC), which acts on the reducing and non-reducing ends to liberate cello-oligosaccharide and cellobiose, while β-glucosidases cleaves cellobiose to liberate glucose for bioethanol production [3]. The major concern in utilising lignocellulose for bioethanol production is the cost of cellulase, which is usually produced from expensive substrates [3, 4]. In fact, it is still expensive despite the huge efforts given to improve its activity and productivity [5]. The utilisation of cheap and readily available agro-waste material in producing cellulase could possibly reduce its production cost and price. In addition, the cost of cellulase could be reduced by four to five times through screening, strain reconstruction and innovation in the production process [6].

To date, commercial cellulases are being produced from fungi due to the high product titre. However, due to the slow growth rate and longer fermentation period of fungi, the cost for its production is yet to be high. Thus, to meet the global demand for cellulase, bacteria cellulase was later used by many researchers due to its high growth rate, versatility, robustness, shorter generation period, genetic stability and the multienzyme complex (MEC) produced [7–11]. Reviews from the literature has also revealed several kinds of bacteria, mainly *Bacillus* [12] and actinomycetes [13] as the efficient cellulase producers. Studies over the years have also focused on using cellulase from mesophilic bacteria to carry out simultaneous saccharification and fermentation (SSF) since their enzyme activity is optimum at a temperature (40 °C) close to that of fermentative yeast (30 °C). However, the awareness on unsustainable dependence on mesophilic yeast, incompatibility in enzymatic hydrolysis and fermentation optimum temperatures and challenges such as low hydrolysis rate, high enzyme loading, high risk of contamination, low thermal stability and incomplete hydrolysis experienced during SSF have prompted recent interest in thermophilic bioprocessing. The thermophilic processing of cellulosic biomass offers several potential benefits including low viscosity, high solubility, low risk of contamination, higher hydrolysis rate, decreased enzyme loading and consumption that leads to overall economics of the process [14]. Due to the needs in high thermal industrial processes with enzyme as biocatalyst and the viability of exploring thermophilic fermentative bacteria in bioethanol production, the demand in thermostable cellulase has increased. Thermophilic cellulase degrading bacteria are said to have a great potential in developing thermostable cellulase for sustainable technologies to efficiently hydrolyse the cellulosic biomass [14]. Nonetheless, there are a very limited amount of thermophilic cellulolytic bacteria isolated from various environments as been previously reported [7, 11, 15–18].

Industries that utilise lignocellulose mostly depend on the use of a single substrate. However, as lignocellulose was being studied to search for its new industrial use due to the advocation of green technology and sustainability, the risk in inaccessibility or scarcity of specific substrate at any point in time could leave a devastating effect on industrial productivity. Combining lignocellulose has helped to improve yield [19, 20] and reduce the production cost [21] of fuel ethanol. Furthermore, mixed feedstocks (agro-waste cocktail) have been reported in the production of fermentable sugars [22, 23]. However, the use of these forms of feedstock to produce bacteria cellulase is considered rare.

For this reason, cellulose degrading bacteria were isolated and characterised from the oil palm empty fruit bunch-chicken manure compost. Since plant biomass cellulose majorly exists in crystalline form [24], it is necessary to apply microcrystalline cellulose (MCC) as a yard stick for screening the cellulase production. Therefore, a quantitative screening approach rather than the conventional CMC screening was adopted in selecting the strain that could best produce cellulase on MCC. The most productive strain was then used to examine cellulase production behaviour on a single and cocktailed agro-waste. Morphological behaviour of the strain and agro-waste was also observed to understand the mechanism of cellulolysis. Furthermore, the location(s) of the enzymes was identified to gain an insight on the enzymatic system of the bacteria strain.

## Materials and Methods

### Composting Feedstocks and Preparation

Shredded oil palm empty fruit bunch (OPEFB) was collected from the Seri Ulu Langat Palm Oil Mill in Dengkil, Selangor, Malaysia. Chicken manure was obtained from the poultry farm, Faculty of Agriculture, Universiti Putra Malaysia. Both feedstocks were utilised for the composting. A total of 80 kg feedstocks were mixed at the ratio of 1:1. Additionally, the required amount of water was added and mixed to achieve 55–65% moisture content. Continuous addition of water was done with turning and mixing. The temperature, pH and moisture content of the compost were also determined during a sampling period of 40 days.

### Cellulolytic Bacteria Isolation

Isolation of bacteria was carried out according to that conducted by Zainudin et al. [25] with some modifications. Briefly, 10 mL of normal saline was added into a 50-mL test tube containing 1 g compost sample. The sample was properly dislodged into the mixture by agitation at 150 rpm for 10 min. One millilitre of mixture was serially diluted and plated onto Luria Bertani (LB) agar containing 1% caboxymethyl cellulose (CMC). The plates were further incubated at 37 and 55 °C for 24 h to allow mesophilic and thermophilic bacterial growth. Repeated picking and re-streaking were carried out on LB-CMC agar incubated at 37 and 55 °C for 24 h, which was then flooded with iodine solution [26]. All strains showing zone of clearance were selected as positive stains and used for further screening.

### Plate Zymogram and Measurement

Cultures of 30 bacteria strain were grown in Luria Bertini (LB) broth until OD_600_ = 0.6. Then 5 μL of each bacteria culture was spot inoculated unto the centre of cellulase medium containing the following (g/L): 1.0 KH_2_PO_4_, 1.145 K_2_HPO_4_, 0.4 MgSO_4_·7H_2_O, 5.0 NH_4_SO_4_, 0.05 CaCl_2_·2H_2_O and 1 mL Nitsch’s trace element solution (2.2 g MnSO_4_, 0.5 g ZnSO_4_, 0.5 g H_3_BO_3_, 0.016 g CuSO_4_, 0.025 g Na_2_MoO_4_ and 0.046 g CoCl_2_) [15]. In the cellulase medium, 1% (*w*/*v*) CMC and 10 g/L bacteriological agar were added. The plates were kept undisturbed for 1 h to allow a complete diffusion, followed by incubation at 37 and 55 °C for 48 h. Later, the plate was flooded with iodine solution for 2 min [26]. The bacteria isolates were examined for CMC hydrolysis by measuring their corresponding halo zones and their hydrolytic capacity was determined by identifying the cellulolytic index using the following expression:

1 $$ \mathrm{EI}=\mathrm{CI}=\frac{\mathrm{Diameter}\ \mathrm{of}\ \mathrm{hydrolysis}\ \mathrm{zone}}{\mathrm{Diameter}\ \mathrm{of}\ \mathrm{colony}} $$

where EI and CI represent enzymatic index and cellulolytic index, respectively [27].

### Inoculum Preparation

Each bacteria strain was cultured up to their log phase on cellulase medium containing 1% CMC. Thereafter, 50 mL of the culture was centrifuged at 10,000×*g* and 4 °C for 10 min. The supernatant was discarded and the cell pellet was repeatedly washed using sterile 0.1% (*w*/*v*) peptone [28]. Cell suspension was set at OD_600_ = 1.0 and used for inoculation process.

### Quantitative Screening on Microcrystalline Cellulose

For quantitative screening, 50 mL of cellulase medium comprising 1% (*w*/*v*) MCC in 100-mL Erlenmeyer flask was introduced with 5% inoculum of bacteria cells. The flasks were incubated at 50 °C for 48 h under shaking at 180 rpm. After that, samples were withdrawn and centrifuged at 10,000×*g* for 10 min at 4 °C. The supernatant was taken and then used as crude enzyme to carry out enzyme activities.

### Characterisation and Identification of Cellulolytic Bacteria

#### BIOLOG Method

Suspension of active bacterial cell culture (grown on NA at 37 °C for 16 h) was prepared in an inoculating fluid (IF-A) at a cell density of 95% transmittance using a BIOLOG turbidimeter following the manufacturer’s instruction. A 100-μL inoculum was dispensed into GEN III microplate wells using a multichannel pipette and incubated at 33 °C for 16 to 24 h after that. Oxidation of various carbon sources and their sensitivity to different chemicals indicated by the reduction in tetrazolium redox dye to purple colour was monitored and recorded on microplate reader. The pattern of oxidation was compared with the BIOLOG database software.

#### DNA Extraction and Molecular Phylogenetic Analysis

Genomic DNA extraction was done using GeneJet genomic DNA extraction and purification kit (Thermoscientific Inc., USA). The genomic DNA was stored at −80 °C and used as a template for 16S rDNA PCR. The 16S rDNA PCR was performed on a T-gradient thermocycler (Labrepco, Germany) with the universal primer of 27F (*5′-AGA GTT TGA TCC TGG CTC AG-3′)* and *1492R (5′-GGT TAC CTT GTT ACG ACT T-3′*). The reaction mixture consists of 12.5 μL REDTaq, ReadyMix PCR Reaction Mix (Sigma-Aldrich, USA), 1 μL of each forward and reverse primers and 35.5 μL of sterile distilled water. The conditions for polymerase chain reaction (PCR) are 98 °C for 5 min initial denaturation, 35 cycles of 95 °C for 30 s, 45.3 °C for 30 s, 72 °C for 90 s denaturation annealing and extension and 72 °C for 8 min final extension of the amplified DNA. The PCR products were purified and sequenced. Sequence similarities were compared using the Basic Local Alignment Search Tool (BLAST) programme on NCBI and 16S rDNA gene sequence homology analysis using Gene Bank data (http://blast.ncbi.nih.gov/Blast). A phylogenetic tree was constructed using the MEGA 6.06 programme through the neighbour joining method. The partial genomic sequence was deposited with the accession number KT799651.

#### Pretreatment of Agro-wastes Biomass and Compositional Analysis

Rice straw was collected from a rice farm of Faculty of Agriculture, Universiti Putra Malaysia. Rice husk was obtained from Bernass Bhd. Sekinchan, Malaysia. Meanwhile, sugarcane bagasse was collected from a sugarcane extractor in a local market located at Taman Seri Serdang, Selangor, Malaysia, whereas an empty fruit bunch was accumulated from Seri Ulu Langat Palm Oil Mill in Dengkil, Selangor, Malaysia. All agro-waste biomass were thoroughly washed using tap water and dried at 60 °C to a constant weight. They were further grinded and sieved to 0.25 mm particle size using a grinder (Retsch SM 200 Rustfrei, Haan Germany). Alkali pretreatment was carried out in a volume ratio of 1:10 (1 g agricultural waste in 10 mL NaOH) with 2% NaOH, followed by autoclaving at 121 °C for 15 min. Samples were washed for several times using water and neutralised to pH 7 with HCl, then dried at 60 °C for 24 h and stored at 4 °C until further use. The analysis of cellulose, hemicellulose and lignin for both the untreated and NaOH pretreated agro-waste materials were determined according to the method described in [29].

#### Cellulase Production on Single Substrate and Agro-waste Cocktail

Cellulase production was carried out using 50 mL cellulase medium in 100-mL Erlenmeyer flask with composition described on ‘Plate Zymogram and Measurement’ paragraph in the ‘Material and Methods’ section containing 1% (*w*/*v*) of each agro-waste biomass. Cellulase production was carried on the untreated rice husk (URH), untreated rice straw (URS), untreated sugarcane bagasse (UBAG), untreated empty fruit bunch (UEFB), pretreated rice husk (TRH), pretreated rice straw (TRS), pretreated sugarcane bagasse (TBAG), pretreated empty fruit bunch (TEFB) and agro-waste cocktail (AWC). Bacterial cells were inoculated at 5%, which were then incubated at 50 °C for 30 h at 180 rpm. One millilitre of sample was taken at every 6 h and centrifuged at 10,000×*g* within 4 °C for 20 min. Meanwhile, the supernatant was used as the crude enzyme.

#### Enzyme Assay

The activity of crude enzyme was determined using the method described by [30]. Carboxymethyl cellulase (CMCase) activity was determined by measuring the reducing sugar released from CMC. A volume of 0.5 mL crude enzyme was put to react with 0.5 mL of 1% CMC in 0.05 M phosphate buffer with pH 7 and incubated at 50 °C for 30 min. Filter paperase (FPase) was determined by assessing the loss of sugar released by filter paper. In this reaction, 0.5 mL crude enzyme was mixed with a 1 × 6-cm (Whatman No. 1) filter paper immersed in 1.5 mL of phosphate buffer with pH 7 and incubated at 50 °C for 1 h. The reducing sugars was measured utilising the DNS method [31]. The reaction was stopped with the addition of 3 mL 3,5-dinitrosalicylic acid (DNS). One unit of enzyme activity is determined by the amount of enzyme required to liberate 1 μmol of reducing sugar per minute under assay condition. For β-glucosidase assay, *p*-nitrophenyl liberated from *p*-nitrophenyl-beta-d-glucopyranoside was spectrophotometrically determined [30]. The reaction mixture was incubated at 50 °C for 30 min. One unit of beta-glucosidase activity was defined by the amount of enzyme used in liberating 1 μmol *p*-nitrophenol per minute under the assay condition. For xylanase activity, the reaction mixture comprised 0.5 mL of 1% birch wood xylan in 0.05 M phosphate buffer pH 7 and 0.5 mL appropriately diluted enzyme, which was incubated at 50 °C for 30 min. Xylanase activity was determined adopting the DNS method. Meanwhile, 1 unit of xylanase activity was determined as the amount of enzyme required to liberate 1 μmol xylose per minute under specified assay condition.

#### Scanning Electron Microscope Analysis

Scanning electron microscopy observation was carried out using the agro-waste biomass and bacteria culture suspension. Five millilitres of *Bacillus licheniformis* 2D55 culture grown on UBAG, TBAG and AWC was centrifuged and processed for scanning electron microscope (SEM) analysis and referred to as cell suspension culture. The agro-waste residue was pipetted after the culture flask was allowed to sit for 20 min and then processed for SEM observation. Samples were mounted after 30 min critical point drying on a metal stubs, which was followed by gold palladium coating adopting the method by Pathan et al. [32]. Scanning electron microscope (JSM 700-151F, JOEL Tokyo, Japan) was used to observe any changes.

#### Localisation of Enzyme

The location of cellulase (CMCase, FPase, β-glucosidase) and xylanase produced by *B. licheniformis* 2D55 was identified on cellulase medium containing 1% (*w*/*v*) of AWC incubated at 50 °C under agitation at 180 rpm for 18 h. A 40-mL culture broth was centrifuged at 10,000×*g* for 10 min at 4 °C. The supernatant was withdrawn and applied as extracellular enzyme. The resulting cell pellet was washed three times with 15 mL 0.05 M phosphate buffer pH 7.0 and then resuspended in a 10-mL final solution of the same buffer. Cell suspension was sonicated at 40% amplitude using a sonication tool (Q Sonica QSS, Newton, CT, USA) for 8 min with 30 s pulse interval. The sonicated cell suspension was then centrifuged at 10,000×*g* for 10 min under 4 °C and the supernatant was withdrawn and used as intracellular enzyme sample while resuspending the cell pellet in 5 mL buffer and using it as a membrane-bound enzyme sample. The remaining AWC was further filtered through a muslin cloth with 0.2 g residue resuspended in 10 mL phosphate buffer and used as the substrate-bound enzyme sample. The enzyme samples used to analyse protein concentration as well as cellulase and xylanase activity were expressed as enzyme activity (U/mg) protein.

#### Protein Concentration Determination

In this study, protein concentration was quantified using Bradford assay [33]. Briefly, 100 μL of enzyme suspension was mixed with 3 mL Bradford reagent (Sigma-Aldrich, St. Louis, MO, USA). The mixture was further incubated for 15 min at room temperature and read at 595 nm against a reagent blank that contains 100 μL 0.05 M phosphate buffer with pH 7 and 3 mL Bradford reagent. Using bovine serum albumin as a standard, the protein concentration was inferred from the standard curve and expressed as milligrammes per milliliter.

## Results and Discussion

### Co-composting of OPEFB and Chicken Manure

**Fig. 1:**
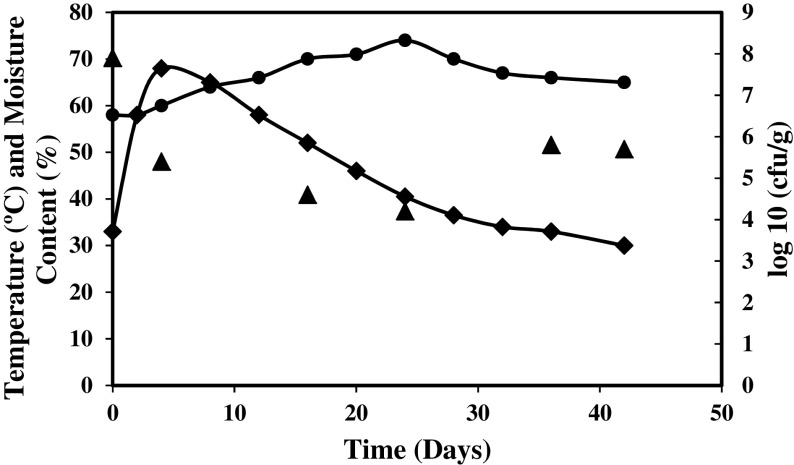
Profiles of temperature, moisture content and bacterial count during composting of OPEFB and chicken manure. *Diamond* indicates temperature, *circle* moisture content and *triangle* bacterial count

In composting, temperature is a crucial factor that determines the progression of this process. Findings on this study have presented that temperature has drastically rose at day 2 with its peak at 68 °C on day 4, which was then slowly declined until the end of composting process (Fig. 1). It was also observed that the high temperature was maintained for a long period (2 to 16 days) within a range of 50–68 °C. This is due to turning and metabolic activities that occurred as a result of microbial degradation. Moisture content during the composting period was found within 58 to 70%, which is in agreement with that conducted by Yahya et al. [1]. The microbial population during composting at thermophilic, mesophilic and cooling temperature were determined. The increase in microbial population at the cooling and maturing stages could be resulted from the availability of simple nutrients that may foster the re-colonisation of bacteria from the environment. Meanwhile, lower microbial count at the thermophilic stage was reported by Jurado et al. [34]. The longest composting period was found to be 136 days with a C/N ratio of 12.1 [34]. In the present study, maturity was attained within 42 days using a C/N ratio of 14.1. This result suggests that OPEFB and chicken manure are the compatible feed stocks for composting.

**Table 1:** Cellulolytic index of bacterial isolates at different stages of composting

Composting stages	Compost period (days)	Temperature	Bacterial isolates	Cellulolytic index (CI)	Relative CI (%)
Initial	0	33	BC1	0.8 ± 0.1	21
Thermophilic peak	4	68	2D55	3.8 ± 0.4^a^	100
			BD2	3.0 ± 0.1	78
			BB2	2.3 ± 0.3	60
Thermophilic	16	52	BB15	2.5 ± 0.5	65
			BB16	2.8 ± 0.1	73
			BB14	2.7 ± 0.6	71
Mesophilic	23	41	B20	2.4 ± 0.3	63
Cooling	36	33	BC22	2.1 ± 0.3	55
Stabilising	42	30	4B32	2.7 ± 0.2	71

^a^Cellulolytic index of isolate 2D55 was 3.8 ± 0.4; this was set at 100% and was used as the basis for calculating relative activity of other isolates

### Screening for Cellulolytic Bacteria

A total of 60 bacterial isolates were obtained. After preliminary screening, 30 isolates were discovered positive on CMC agar with 9 of them having a high cellulolytic index (CI) ≥ 2 (Table 1). The maximum cellulolytic index of 3.8 with 100% relative activity was produced by isolate 2D55, followed by BD2 and BB16 with relative activity of 78 and 73%, respectively. Majority of isolates at the thermophilic stage was observed with a higher CI than isolate at mesophilic, cooling and stabilising temperatures. This could be attributed to the active degradation occurred at such temperatures. According to [35], higher cellulase activity was observed at thermophilic temperatures for all treatments during the composting of green waste, earthworm cast and zeolite. The three best isolates (2D55, BB16 and BD2) were selected to determine their cellulase productivity when grown on MCC as a carbon source.

### Quantitative Screening for Cellulase Production on Microcrystalline Cellulose

**Fig. 2:**
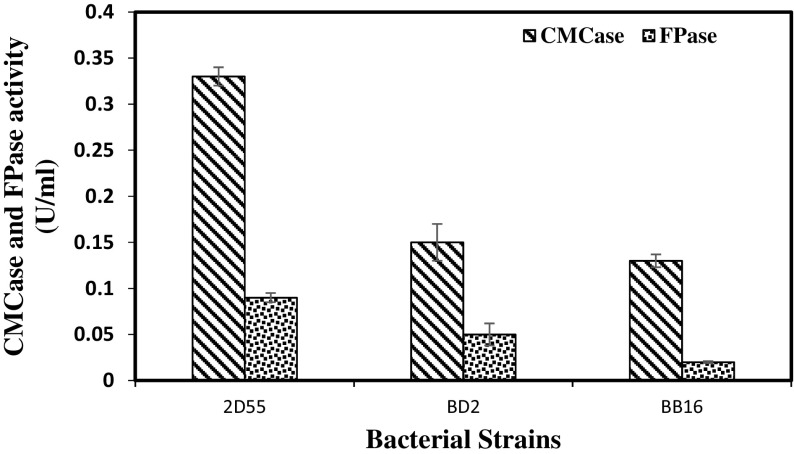
Quantitative screening for cellulase production by bacterial strains grown on microcrystalline cellulose. Values are means of (*n* = 3), ±SD (*vertical bars*)

In establishing the potential of cellulase production, isolates 2D55, BB16 and BD2 were grown in cellulose medium with microcrystalline cellulose (MCC) as the carbon source (Fig. 2). Isolate 2D55 had produced maximum CMCase activity at 0.33 U/mL and FPase activity at 0.09 U/mL after 24 h, while BD2 and BB16 have produced CMCase at 0.15 and 0.13 U/mL, respectively after 30 h. CMCase has demonstrated significantly higher activity compared to FPase in this study. The high CMCase activity observed in this study is similar to that previously reported by the studies from [14, 36]. There are very few reports on cellulase production by *Bacillus* sp. grown on microcrystalline cellulose as the sole carbon source [14, 15] (Table 2). However, the CMCase from isolates 2D55, BD2 and BB16 are yet to be compared in cellulase production with the chemically defined medium reported by [13]. Rastogi et al. [14] reported that CMCase (0.12 U/mL) and FPase (0.03 U/mL) were produced by thermophilic *Bacillis* sp. on the 9th and 8th days, respectively, when microcrystalline cellulose was used as a substrate, while *Geobacillus* sp. was reported to produce 0.13 U/mL CMCase and 0.04 U/mL FPase activity on the 7th and 8th days. Interestingly, the maximum cellulase production achieved in this study is between 18 and 36 h among the isolates. Cellulase production at a high rate by isolate 2D55 was identified as remarkable. Findings from this study are in contrast with the previous research, which shows that moderately thermophilic *B. licheniformis* B-41361 has produced CMCase on glucose and not on CMC and MCC as the carbon source [37]. Other thermophilic *Bacillus* sp. including *Anoxybacillus* and *Brevibacillus* sp. were reported to utilise cellulose as carbon source [17]. Isolate 2D55, which was examined with maximum cellulase titre among the isolates, was selected for further studies.

**Table 2:** Cellulase production from some thermophilic bacteria grown on microcrystalline cellulose

CMCase (U/mL)	FPase (U/mL)	Temperture (°C)	Bacterial isolates	Reference
0.33	0.43	50	*Thermobifida fusca*	[13]
0.058	0.043	60	*Geobacillus* sp*.*	[15]
0.12	0.03	60	*Bacillus* sp.	[14]
0.13	0.04	60	*Geobacillius* sp.	[14]
0.33	0.09	50	*Bacillus licheniformis* 2D55	This study

Results presented were performed under unoptimized conditions

### Characterisation and Identification of Bacteria Isolates

The bacteria isolates were characterised by utilising carbon source and chemical sensitivity following the BIOLOG GENIII method (Table 3), while the morphological and physiological characterisation of isolate 2D55 was done as indicated in Table 4. The isolates were found utilising dextrin, d-maltose, d-trehalose, cellobiose, d-gentiobiose, sucrose, d-turanose, l-arginine and l-glutamic acid with inhibition displayed by vancomycin. Isolates 2D55 and BB16 have demonstrated tolerance towards lithium chloride, sodium lactate and guanidine HCl with isolate BD2 showing a low tolerance. Interestingly, the ability of these three isolates to utilise cellobiose is able to influence the induction of cellulase. On the other hand, previous studies on *Anoxybacillus* 527 showed a higher production of cellulase on cellobiose compared to MCC [17]. Their ability to oxidise lithium chloride may also suggest their potential application in the biodegradation of lithium contaminated environment. The results obtained from the BIOLOG tool provide an easier and faster method in measuring bacterial phenotypic characteristics. It can be seen in Table 4 that isolate 2D55 is a gram-positive aerobic bacillus. It demonstrated the ability to grow within pH 3–8 in the presence of 2–8% NaCl with temperatures ranging from 30 to 60 °C. Physiological characterisation using the BIOLOG tool was also reported in a thermophilic cellulase producing *Bacillus* sp. strain C 1 isolated from cow dung [38].

**Table 3:** Biochemical characterisation of cellulolytic isolates using BIOLOG GEN III

Parameters	Selected cellulolytic isolates										
Carbon	2D55	BD2	BB16	Carbon/acid	2D55	BD2	BB16	Acid, antibiotic, pH, salt	2D55	BD2	BB16
Dextrin	+	+	+	d-Arabitol	−	−	nd	Gelatin	−	+	+
d-Maltose	+	+	+	Glycerol	−	nd	+	d-Galactose	nd	−	+
d-Trehalose	+	+	+	Gelatin	−	+	+	Propionic acid	−	−	−
d-Cellobiose	+	+	+	d-Galactose	nd	−	+	Acetic acid	−	−	+
Gentiobiose	+	+	+	l-Rhamnose	−	−	+	l-Lactic acid	nd	+	+
Sucrose	+	+	+	l-Alanine	+	nd	+	Vancomycin	−	−	−
d-Turanose	+	+	+	l-Arginine	+	+	+	Rifamycin	−	−	−
d-Raffinose	nd	+	+	l-Glutamic acid	+	+	+	Guanidine HCL	+	nd	+
α-d-Lactose	−	nd	+	Pectin	nd	+	−	Lithium chloride	+	nd	+
d-Mellibiose	nd	nd	+	d-Galactouronic acid	+	+	−	Sodium lactate	+	+	+
α-d-Glucose	+	+	+	d-Gluconic acid	+	+	+	Potassium tellurite	+	nd	+
d-Mannose	nd	+	+	Mucic acid	+	+	+	Sodium butyrate	+	+	+
d-Fructose	nd	+	+	l-Lactic acid	nd	+	+	pH 6	+	+	+
d-Galactose	nd	−	+	l-Citric acid	nd	nd	+	pH 5	+	+	+
l-Rhamnose	−	−	+	d-Arabitol	−	−	nd	1% NaCl	+	+	+
d-Sorbitol	+	+	−	Glycerol	−	nd	+	4% NaCl	+	+	+
d-Mannitol	+	+	−					8% NaCl	+	+	+

Tentative identity: 2D55 *Bacillus* sp., BD2 = *Bacillus subtilis*, BB16 = *Bacillus badius*

*nd* undetermined, + = positive reaction, − = negative reaction

**Table 4:** Morphological and physiological characterisation of isolate 2D55

Parameters	Isolate 2D55 results
Cell morphology	
Shape	Rod
Colony	
Colour	Whitish
Surface	Rough, hair-like outgrowth
Margins	Undulte to fimbrate
Size	2–3 mm in diameter
Gram stain	+
Spore	+
Motility	+
Aerobic growth	+
Anaerobic growth	−
Growth positive at	
30 °C	++
50 °C	+++
60 °C	++
Growth in medium pH	
pH 3	++
pH 5	+++
pH 6	+++
pH 8	+++
pH 10	+
Growth in NaCl	
2%	+++
4%	+++
8%	+++
10%	+
12%	−

− = negative, + = positive, ++ = moderate, +++ = high

### Identification of Isolate 2D55 Using 16S rDNA

**Fig. 3:**
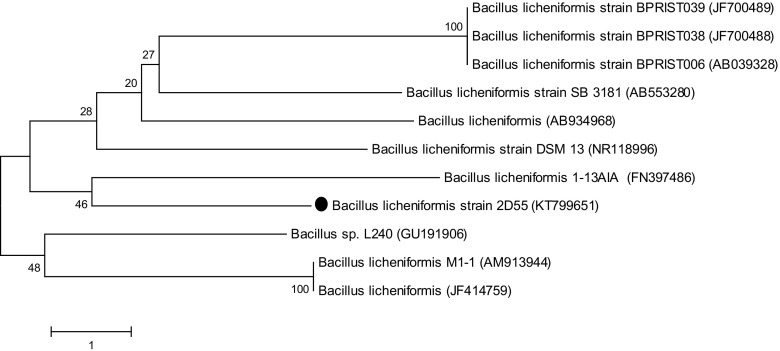
Phylogenetic dendogram showing relatedness between 16s rRNA of the isolated bacterium and related bacteria strains from gene data base using the neighbour joining method

The nucleotide sequence of PCR product was compared with other sequences of 16S rDNA in the Gene Bank database by BLASTN and the accession number of KT799651 was obtained from the NCBI (Fig. 3). The phylogenetic tree generated using the neighbour joining method showed a connection between the species of *B*. *licheniformis* and *Bacillus* sp. The strain also showed a 99% similarity with *B. licheniformis* DSM13 [39]. Additionally, 2D55 presented 99% similarity with *B. licheniformis* 1-13AI, a gram-positive, thermophilic, aerobic, halotolerant bacterium isolated from human faeces [40] and strain *B. licheniformis* M1-1 isolated from enrichment cultures of composting materials at 50 °C. Therefore, strain 2D55 was considered to be highly related to different strains of *B. licheniformis*. Strains of *Bacillus* sp. that belong to *Firmicutes* are known to play a major role in cellulolytic and hemicellulolytic activities during lignocellulosic degradation at the thermophilic stage of composting [25].

### Effects of Single/Cocktail Agro-waste on Cellulase Production by *B. licheniformis* 2D55

The use of agro-waste materials as the carbon sources for cellulase production has both economic and environmental advantages. The effects of using agro-waste for cellulase production were verified on rice straw, rice husk, sugarcane bagasse and empty fruit bunch. *B*. *licheniformis* 2D55 was observed to utilise both untreated and pretreated agro-waste materials for cellulase production (Table 5). For cellulase production on untreated agro-waste, the best carbon source was UBAG (CMCase 0.102 U/mL, FPase 0.160 U/mL), which was followed by the URS (CMCase 0.016 U/mL, FPase 0.018 U/mL). The minimum cellulase production (CMCase 0.004 and 0.012 U/mL) was observed on UEFB and URH, respectively. FPase activity was not produced on UEFB and URH. The cellulosic composition analysis showed a varying level of different agro-waste materials. The cellulose composition of untreated agro-waste was observed in the trend of EFB > RS > RH > BAG, while hemicellulose and lignin content showed a different trend of RH > EFB > BAG > RS.

**Table 5:** Effect of agro-waste as carbon source for cellulase production by *B. licheniformis* 2D55

Agro-waste	CMCase	FPase	Optimum incubation time (h)	Cellulose (%)	Hemicellulose (%)	Lignin (%)	Others (%)
Untreated							
URH	0.012 ± 0.012	nd	18	37.13 ± 0.13	34.81 ± 0.22	18.20 ± 0.79	12.86 ± 0.38
URS	0.016 ± 0.004	0.018 ± 0.001	18	38.47 ± 0.39	25.08 ± 0.50	9.82 ± 1.23	26.63 ± 0.35
UBAG	0.102 ± 0.010	0.160 ± 0.010	24	33.06 ± 2.03	26.18 ± 1.73	13.80 ± 1.43	26.96 ± 0.35
UEFB	0.004 ± 0.020	nd	12	40.40 ± 1.53	30.52 ± 0.94	18.10 ± 2.03	10.98 ± 0.58
Pretreated							
TRH	0.150 ± 0.004	0.019 ± 0.010	18	65.15 ± 0.15	25.43 ± 1.28	9.08 ± 0.98	7.34 ± 0.62
TRS	0.081 ± 0.020	0.020 ± 0.002	24	50.02 ± 0.72	24.58 ± 1.03	7.81 ± 0.53	17.59 ± 1.08
TBAG	0.090 ± 0.010	0.050 ± 0.005	18	56.19 ± 1.11	22.32 ± 0.19	8.20 ± 1.44	13.29 ± 0.63
TEFB	0.080 ± 0.015	0.010 ± 0.003	18	48.22 ± 2.53	28.64 ± 1.27	13.64 ± 0.87	13.50 ± 0.88
Mixed							
UBAG + TRH (MAW)	0.380 ± 0.070	0.220 ± 0.140	24	44.20 ± 1.05	24.80 ± 0.87	12.31 ± 1.36	18.69 ± 2.01

Values are means of (*n* = 3), ±SD (vertical bars)

*RH* rice husk, *RS* rice straw, *BAG* sugarcane bagasse, *EFB* empty fruit bunch, *UBAG* untreated bagasse, *TRH* treated, *MAW* mixed agro-waste

After NaOH pretreatment, the maximum cellulase production was observed on TRH (CMCase 0.150 U/mL, FPase 0.019 U/mL), followed by TBAG (CMCase 0.090 U/mL, FPase 0.50 U/mL). The cellulase production recorded on TRS (CMCase 0.081 U/mL, FPase 0.020 U/mL) is comparable to that recorded on TEFB (CMCase 0.080 U/mL, FPase 0.010 U/mL). Since untreated BAG showed the highest FPase activity with TRH demonstrating the highest CMCase activity; hence, the combination of both substrates at 1:1 ratio was used as agro-waste cocktail (AWC) in cellulase production. Cellulase production was found to be remarkably increased on AWC (CMCase 0.380 U/mL, FPase 0.220 U/mL). Based on the maximum cellulase titres obtained on the agro-waste tested, it was observed that AWC has improved CMCase and FPase activity by 3.7 and 1.4 times better than UBAG, while a 2.5- and 11.5-fold increase in CMCase and FPase was observed in comparison with TRH. Similarly, the pretreatment of RH has significantly improved CMCase by 12.5-fold. The application of NaOH pretreatment on agro-waste biomass has improved cellulose and decreased the lignin composition of all carbon sources tested. The cellulose components decrease in the order of TRH > TBAG > TRS > TEB, hemicellulose TEFB > TRH > TRS > TBAG and lignin TEFB > TRH > TBAG > TRS.

Table 5 indicates that different agro-waste materials display different capacity for cellulase (CMCase and FPase) induction. Besides, cellulase production was notably increased on the pretreated substrates compared to the untreated one with the exception of TBAG. The high cellulase titres (FPase) observed with UBAG may be attributed from some growth promoting factors that influence the metabolism of bacteria. Lignocellulosic materials contain various proteins, amino acids and mineral elements aside from cellulose hemicellulose and lignin [41]. Gaur et al. [11] compared the cellulase production on untreated sugarcane bagasse, rice husk, rice bran, wheat bran and maize bran by a thermophilic *Bacillus vallismortis* RG-07. Based on their observation, sugarcane bagasse is the most suitable candidate for cellulase production, followed by rice husk and rice bran. Contrary to this, Sadhu et al. [38] reported that thermophilic *Bacillus* sp. supported lesser cellulase production on untreated sugarcane bagasse than on newspaper and orange scale. These observations could be due to strain behavioural differences, substrate preferences, different media compositions and processing conditions used within the studies. The ability of *B. licheniformis* 2D55 to produce higher titres of FPase on UBAG suggests its suitability in industrial production without incurring additional cost on the pretreatment.

The pretreatment of agro-waste material generally increases degradation, which could be due to solubilisation of lignin and hemicellulose as well as morphological and structural changes in the cellulosic fibre. In this study, the high cellulase titre (CMCase) recorded on TRH is similar to a recent observations made by Oke et al. [42] where *Bacillus auerius* S5.2 had produced maximum cellulase on dilute acid pretreated rice husk compared to dilute acid treated EFB and oil palm mesocarp fibre (OPF). Similarly, lower cellulase titres observed on TBAG is in line with previous study conducted by Rahnama et al. [41] that shows a lower cellulase production by *Trichoderma harzanium* SRN3 on NaOH pretreated rice straw than on untreated rice straw. This could also suggest that several unknown components involved in cellulase induction were affected by the pretreatment or there could be a partial disruption of crystalline cellulose portion in the BAG. Nonetheless, Brijwani and Vadlani [43] argued that the changes in hemicellulose and lignin as well as the modification of cellulose might counter the positive effects of alkali pretreatment. Furthermore, maximum cellulase production observed in AWC was supported by the studies carried out by Oke et al. [42] and Yang et al. [7], which reported higher titre of cellulase on mixed agrowaste (EFB, OPF, RH) and (CMC, wheat bran), respectively. Lower cellulase was produced when only CMC was used as the carbon source in the latter study. Surprisingly, cellulase production on the pretreated agro-waste materials was composition-related as CMCase titre and cellulose composition showed similar profiles. On the other hand, different scenario was observed on AWC. Although the pretreatment of lignocellulosic material was reported to increase the size of pore, pore volume separation of lignin from cellulose and hemicellulose has thereby improved the accessibility for bacterial degradation [44-46]. The results obtained in this study suggest that other nutritional factors (proteins, accessible mineral elements and possibly some sugars) present in UBAG might have given a greater influence on high cellulase productivity than cellulose composition. This is the report establishing the improvement of cellulase production by supplementing pretreated rice husk with untreated sugar cane bagasse. This strategy could reduce the pretreatment cost for producing cellulase by using agro-waste cocktail. Furthermore, the use of agro-waste cocktail for cellulase production will provide substrate security and maximise available substrates. Thus, the extreme dependency on single substrate that might result in short of supply can be avoided. Studies on other applications of combined agro-waste biomass have resulted in high product yields with respect to bioethanol production [44, 47] and saccharification [48, 49].

**Fig. 4:**
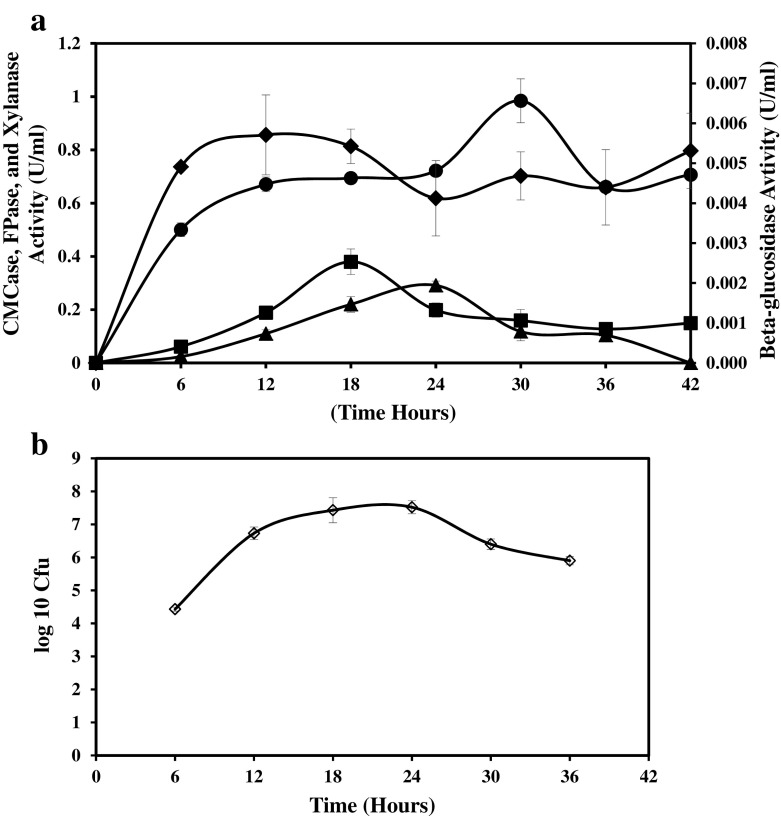
**a** CMCase, FPase, xylanase, β-glucosidase production by *Bacillus licheniformis* 2D55 grown on agro-waste cocktail (AWC) containing a combination of untreated bagasse and pretreated rice husk ratio 1:1. Values are means of (*n* = 3), ±SD (*vertical bars*). *Circle* indicates xylanase activity, *square* CMCase activity, *triangle* FPase activity and *diamond* beta-glucosidase activity. **b** Growth profile of *B. licheniformis* 2D55 grown on agro-waste cocktail. Values are means of (*n* = 3), ±SD (*vertical bars*)

Figure 4 presents the enzyme production and growth profile of *B. licheniformis* 2D55 on AWC. Apart from CMCase and FPase, *B. licheniformis* 2D55 can also secrete xylanase and β-glucosidase. Maximum CMCase and FPase were identified at 18 and 24 h, while xylanase and β-glucosidase have reached their optimum titres at 12 and 30 h, respectively. Apparently, significant interest has been drawn towards xylanase due to its potential industrial applications in the hydrolysis of xylan in lignocellulose [50]. The high xylanase produced could suggest that the mechanism of cellulase production by *B. licheniformis* 2D55 might be obtained using xylanase to degrade the hemicellulose network in the agro-waste biomass, which could provide better accessibility to cellulose. Previous studies [51, 52] showed that a strain of *B. licheniformis* SVD1 grown on birch wood xylan has produced a multi enzyme complex (MEC) comprising several enzymes that include cellulase, xylanase, mannanase and pectinase. *B. licheniformis* 2D55 reached logarithmic phase at 6–12 h after its growth has maintained stationary within 14 to 24 h and later declined until the end of fermentation. Bacterial cells still remain metabolically active as suggested by the maximum CMCase and FPase observed at stationary phase, while optimum xylanase was produced at death phase. Previously, Rastogi et al. [14] reported a maximum CMCase production at the death phase by thermophilic *Bacillus* DUSELR13 with *Geobacillus* WSUCRF1 producing a maximum CMCase and FPase at the end of stationary phase. These results suggest that the regulatory mechanism in *B. licheniformis* 2D55 is different from other bacterial species. In addition, *B. licheniformis* 2D55 could also be producing some membrane-bound enzymes that might have assisted in increasing cellulase activity at the stationary phase. The low cell yield observed during enzyme production could be resulted from the repression of some growth proteins during thermophilic fermentation. However, it is argued that thermophiles have been shown to experience low cell yield in association with enzyme production [14].

### SEM Observation

**Fig. 5:**
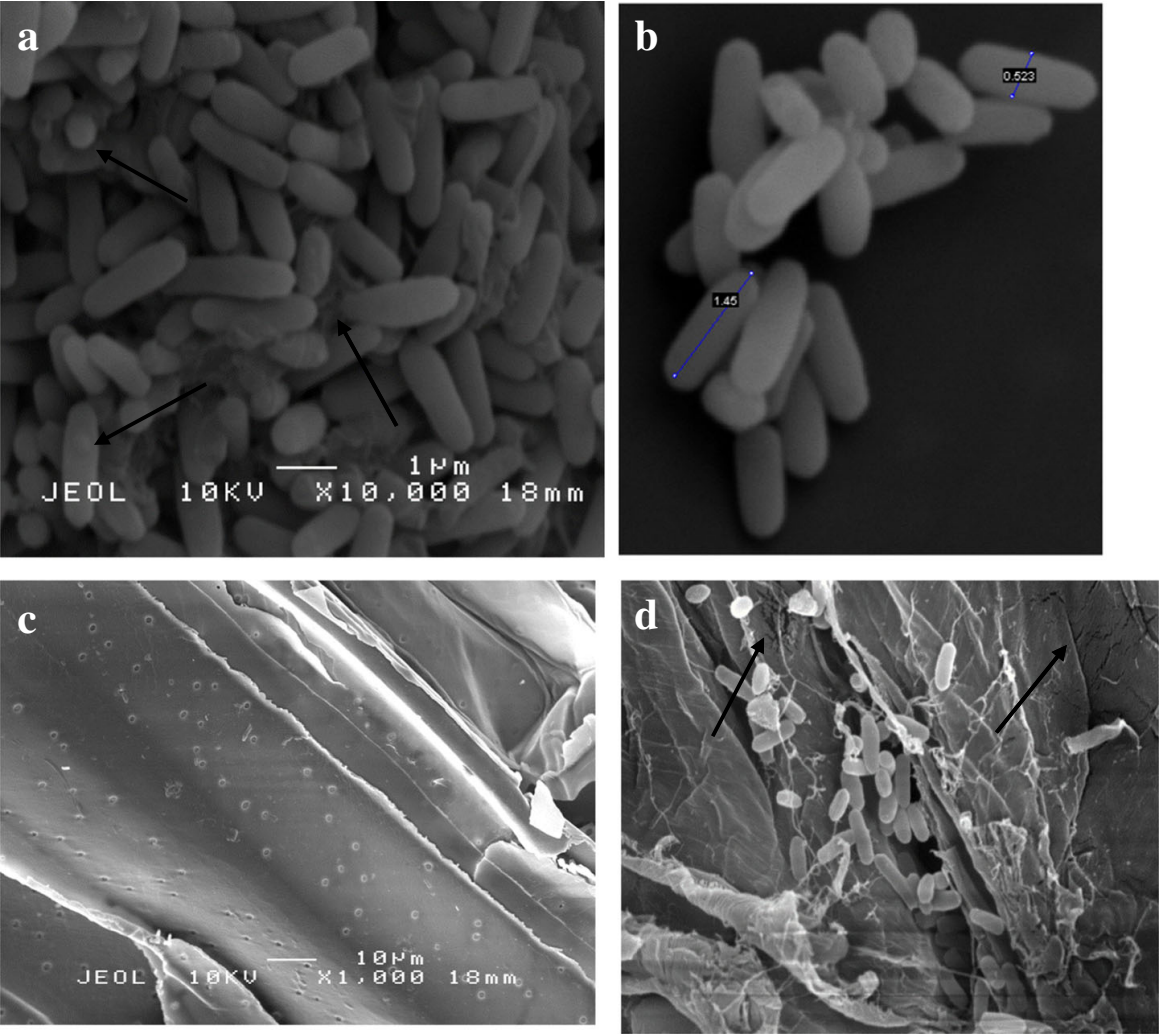
SEM image of cell suspension sample from culture grown on UBAG (**a**, **b**), UBAG before inoculation (**c**) and residue from culture grown on UBAG (**d**)

**Fig. 6:**
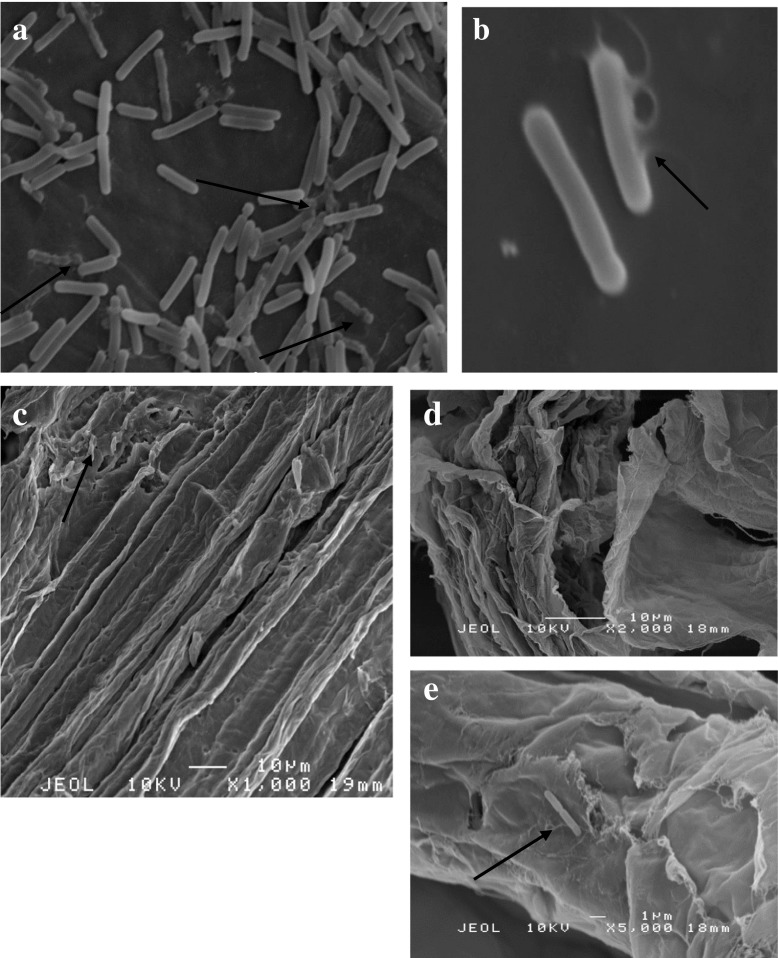
SEM image of cell suspension sample from culture grown on TBAG (**a**, **b**), TBAG before inoculation (**c**) and residue from culture grown on TBAG (**d**, **e**)

**Fig. 7:**
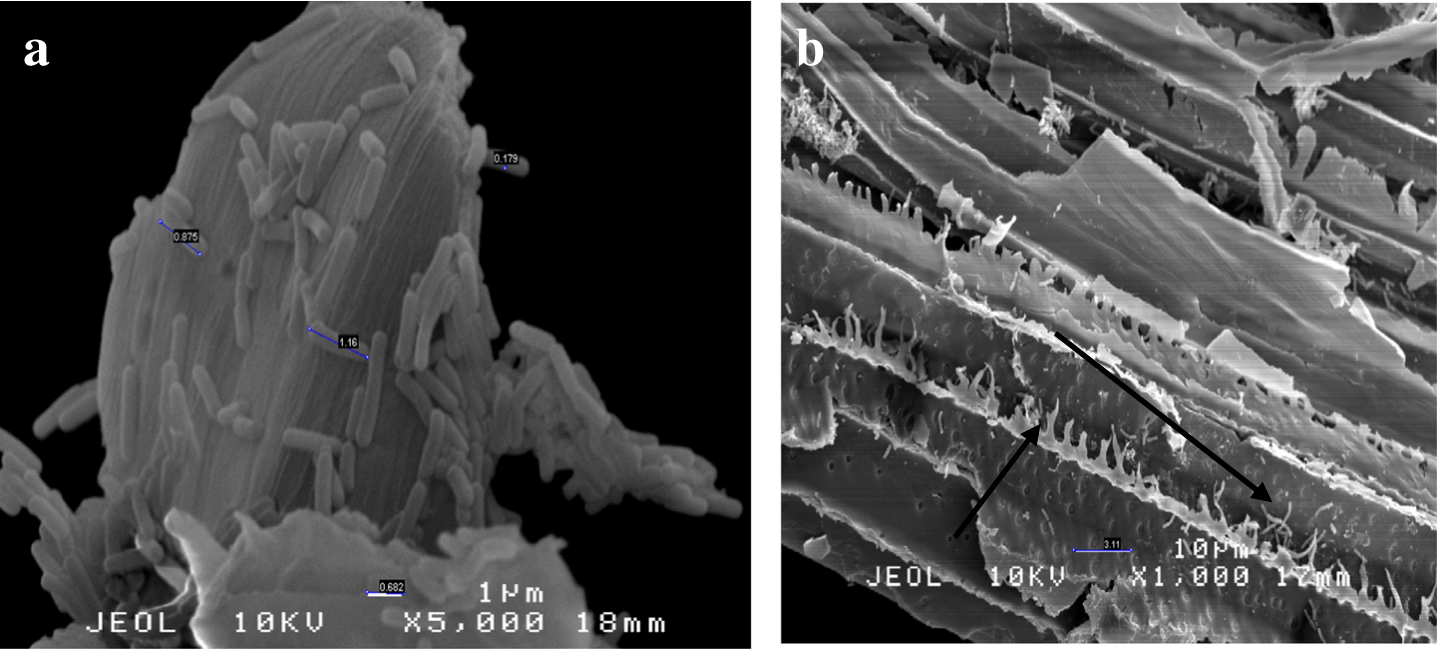
SEM image of AWC residue of culture grown on AWC (**a**) and AWC residue degradation (**b**)

SEM analysis was employed to understand the behaviour of *B. lichermnifmis* 2D55 especially on sugarcane bagasse and the reason cellulase production has not improved despite the pretreatment of sugarcane bagasse. The scanning electron microscopy of *B*. *licheniformis* 2D55 identified the cells as rod-shaped, motile, spore-forming bacteria. Meanwhile, their sizes were ranged from 1.45 to 0.863 μm by 0.53 to 0.156 μm. As shown in Fig. 5a, b, there are a large number of healthy cells in the cell suspension culture grown on UBAG. The cells were noticed to have secreted a network of web-like structures woven around the cells. The cell surface was observed to be rough and woolly. On the other hand, the UBAG (before inoculation) displayed a smooth, rigid and structurally intact surface (Fig. 5c). The morphology of the UBAG is similar to that previously reported [53]. Several healthy cells were attached to the UBAG residue taken from the bottom of the flask of cells grown on UBAG and the residue showed dislodges structures and cracks as a result of degradation by the bacteria (Fig. 5d). However, in Fig. 6a, which displays cell suspension of culture grown on TBAG, a more slender, unhealthy, deformed and lysed cells were observed with a leakage in the cell membrane (Fig. 6b). Figure 6c shows severe surface modification due to rough, rugged, uneven and cracks walls on the TBAG (before inoculation). Also, there is a little or no cells found attached on the TBAG culture residue (Fig. 6d, e). This demonstrates that there are many bacterial cells occupying the liquid suspension compared to the residue, hence blocking the bacteria from making a successful attachment with the substrate for cellulase induction, although the pretreatment has opened lignocellulose structure by breaking down the strong bond between cellulose, hemicellulose and lignin for better accessibility for microbial degradation. Regrettably, chemical pretreatment may also release some additional products including the products from lignin degradation such as aromatic, phenolic, polyaromatic and aldehydic compounds [54]. These products are able to inhibit bacterial growth and metabolism. For instance, phenolic compounds can cause the division and loss of integrity on the cell membrane. Consequently, cell growth, respiration and sugar assimilation are reduced [55]. This could be also related to the reduction of cellulase production observed on the TBAG substrate. Nevertheless, the bacterial cells appeared vibrant and healthier on AWC residue (Fig. 7a). The cells are mass and firmly attached compared to cells on UBAG and TBAG. A closer observation showing cell population, modification and degradation of AWC is presented in Fig. 7b. A similar SEM image was also used to support variations in cellulase production by a thermophilic *Brevibacillus* JXL when grown on cellulose, glucose and cellobiose [5]. According to their study, a great number of cells were found in close attachment of the cellulose residue and cell suspension. They also observed a much denser cellulosomal-like structures and higher cellulase activity when the bacteria were cultivated in glucose compared to cellobiose. Moreover, the cellulosomal structure was found in relation to cellulase production by *Thermobifida fusca* [56], *Bacillus megaterium* [57] and *B. licheniformis* [51]. The SEM observation in the present study does not revealed any visible cellulosomal structure; thus, it cannot be concluded that cellulosomal structure is associated with the high cellulase activity on AWC. Based on the SEM image in this study, it can be concluded that *B. licheniformis* 2D55 had a better growth and attachment when grown on AWC, which resulted in a higher cellulase production.

### Localisation of Enzyme Produced by *B. licheniformis* 2D55

**Fig. 8:**
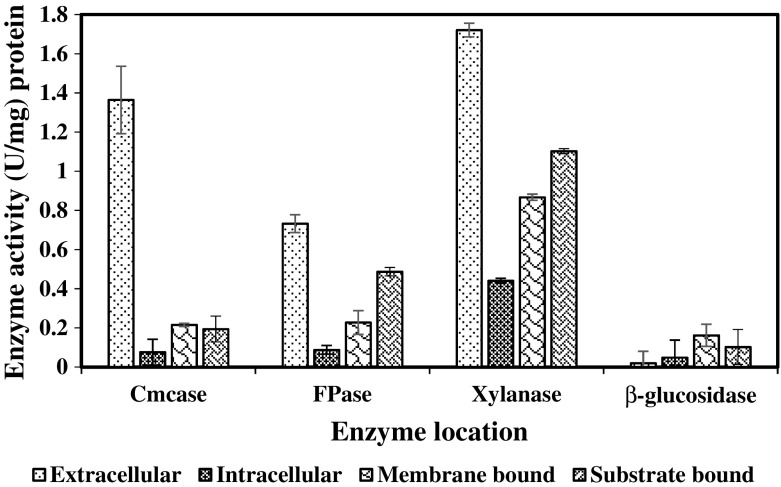
Localisation of enzyme produced by *B. licheniformis* 2D55 grown on mixed agro-waste material

A study was conducted to identify the location of cellulase, xylanase and β-glucosidase activity produced by *B. licheniformis* 2D55 grown on AWC. This is to determine the expression patterns of enzyme in relation to cellular location in the bacteria (Fig. 8). The activity of CMCase, FPase and xylanase was found to be largely extracellular with the highest occurred in xylanase with β-glucosidase being the predominantly membrane bound. This is in agreement with the earlier production profile of enzymes in Fig. 4a where xylanase activity was observed higher than the cellulase enzyme extracellularly produced. It was argued that for cellulose degradation to be most efficient, there must be a direct contact between bacteria and substrate and that the enzyme must be secreted into the fermentation medium [58, 59]. The activity of CMCase, FPase, xylanase and β-glucosidase was also detected as intracellularly and membrane bound; however, the expression pattern was lower compared to the extracellular secretion. So far, cell-bound cellulase and xylanse have been scarcely reported in *Bacillus* sp. The findings of the current study have showed some dissimilarities with a previous report [60] in which CMCase, FPase and xylanase produced by *Bacillus subtilis* was absent in the cell debris. Cell-bound β-glucosidase has also been reported in the previous studies on *Bacillus* strains [60, 61]. In addition, the enzymes were still highly bounded to the membrane than intracellular location. High activity (CMCase, FPase and xylanase) was yet detected on the AWC residue even after separation. This could be resulted from the combination of extracellular enzyme residue, membrane-bound enzyme of the remaining bacterial cells in the AWC residue and those originally bounded to the substrate during cellulase production. This result corroborates the earlier SEM observation on Fig. 7a where high cell mass and close attachment of bacterial cells to the AWC were observed. Extracellular enzymes are less prone to proteolysis and can be easily extracted and purified than intracellular enzymes, hence having a more promising implication. However, cell-bound enzymes are also efficient in assisting degradation, which can potentially enhance the production of cellulase.

## Conclusion

In this study, a gram-positive rod, spore-forming, aerobic, halotolerant, motile cellulase degrading thermophilic *B. licheniformis* 2D55 was successfully isolated from the thermophilic stage during the composting of OPEFB-chicken manure. The strain exhibited higher level of cellulase when grown on microcrystalline cellulose (MCC) compared to other isolated strains. Although a significant cellulase activity was recorded on MCC, the characteristic productivity of individual cellulase has shown a unique nature on agro-waste materials. For example, *B. licheniformis* 2D55 had produced higher FPase than CMCase on untreated sugarcane bagasse, while a lower FPase with higher CMCase was recorded on NaOH pretreated rice husk. Apparently, both CMCase and FPase demonstrated a remarkable improvement on agro-waste cocktail of the untreated sugarcane bagasse and pretreated rice husk. This technique has a great potential in obtaining suitable substrates to improve individual cellulase production on agro-waste materials. *B. licheniformis* 2D55 presented a synergistic multi-lignocellulolytic enzymes including CMCase, FPase, xylanase and β-glucosidase, which makes it potentially suitable for synergistic degradation of complex lignocellulosic substrate, a requirement in biofuel industry. Additionally, *B*. *licheniformis* 2D55 was also reported to possess active thermostable cellulase up to 60 °C during the previous study [62].
